# Effects of different straw returning amounts and fertilizer conditions on bacteria of rice’s different part in rare earth mining area

**DOI:** 10.1038/s41598-023-27553-z

**Published:** 2023-01-09

**Authors:** Shulan Jin, Yizong Huang, Chengxu Dong, Yijun Bai, Huahua Pan, Zhongjun Hu

**Affiliations:** 1 Shangrao Normal University, Shangrao, 334000 China; 2 Scientific Research and Monitoring Institute of Environmental Protection, Ministry of Agriculture and Rural Areas, Tianjin, 300191 China

**Keywords:** Soil microbiology, Microbiology techniques

## Abstract

Pot experiments were conducted to explore the effects of different rice straw returning soil on the community structure and function of bacteria in rice root, rhizosphere, leaf and phyllosphere under 7 conditions of rice straw combined with different fertilizers respectively. The results showed that: rice straw returning in different ways increased the content of soil pH and K, and reduced the accumulation of N, P and organic matter in soil, and different rice straw returning ways had different effects; rice straw returning reduced dry weight of rice grain, 2% of rice straw returning reduced rice grain greater than that of 1% rice straw returning; The reduction of NP combined fertilization is greater than that of NK combined fertilization and NPK combined fertilization. Except for the decrease of chao_1 index in rice root at maturity, rice straw returning significantly improved the abundance, diversity and evenness of bacteria in rice root, rhizosphere, leaf and phyllosphere. Rice straw returning increased the content of REEs in rice, and 2% of rice straw returning soil increased rare earth element (REE) content in rice grain greater than that of 1% rice straw returning soil. Different ways of rice straw returning soil reduced the abundance of Bacillus, while the abundance of *Exiguobacterium* in rice leaves was hundreds of times higher than that of the control group, and the genus in leaves was dozens of times higher than that of the control group, 2% of rice straw returning soil increased the abundance of harmful bacteria and pathogens of *Acidovorax*, *Clostridium *sensu stricto, *Citrobacter*, *Curtobacterium*, and 1% of rice straw returning soil promoted the abundance of nitrogen fixing bacteria, plant growth-promoting bacteria, stress resistant bacteria such as *Lactobacillus*, *Azospira*, *Acinetobacter*, *Bradyrhizobium* and *Acidocella*; Environmental factors such as available P, organic matter, total nitrogen, nitrate nitrogen, rare earth element content in rice roots, available K and soil moisture are important factors affecting the community structure of bacteria in rice roots, rhizosphere, leaf and phyllosphere at tillering stage of the rice. pH, REE content in rice roots, shoots, organic matter, total nitrogen, nitrate nitrogen and soil moisture content are the main environmental factors affecting the community structure of bacteria in rice roots, rhizosphere, leaf and phyllosphere at maturity stage of rice. 2% rice straw returning soil promoted the formation of harmful bacteria, which may be an important reason for its significant reduction in the dry weight of rice grains.

## Introduction

Rare earth elements (REEs) are widely used in industry, agriculture, medicine and other industries because of their unique physical and chemical properties. They have become strategic resources with international influence. It is reported that, the global output of rare earth mines increased from 110,000 tons to 210,000 tons from 2012 to 2019^1^; In 2019, China’s rare earth mineral output was 132,000 tons, accounting for 63% of the global rare earth mineral output, and the output of ionic rare earth accounted for about 90% of the world^1–5^. The excessive production of rare earth minerals causes a large number of REEs to enter the soil, water and crops in and around the mining area, the average value of soil rare earth content in typical rare earth mining areas in Jiangxi Province is 976.94 mg/kg, which is 4.53 times and 5.09 times of the background value of soil rare earth content in Jiangxi Province and the whole country^6^; The concentrations of REEs in river water and well water of the mining area are 55.72 mg/L and 0.033 mg/L, which are 8974.7 times and 10.55 times of the concentration in the control area respectively. The rare earth content of many vegetables in the mining area is 10–20 times higher than the national food limit standard^7^. After exogenous REEs enter the environment, they are harmful to plants, animals and microorganisms^8,9^. REEs in the environment enter the human body through the food chain and accumulate in the body, which threatens the health of residents in the mining area.

Rice straw returning affected soil physical and chemical properties. Ionic rare earth ores are mainly distributed in the south of China centered in Ganzhou, Jiangxi Province. This region is the main rice production area in China, producing hundreds of millions of tons of rice straw every year. Studies have shown that rice straw returning soil can reduce the soil bulk density of 0–10 cm plough layer by 0.17–0.25 g/cm^3^, significantly improving the total porosity and capillary porosity^10^; the rice straw will release alkaline metals, anions and organic components after soaking in water. The release of organic acids in the early stage of rice straw decomposition will cause a weak acidification process of the pH in the surface aqueous solution. After the rice straw is decomposed, there will be an alkalization process, which causes the increase of pH^11^. Rice straw contains 56.5% of organic matter, 1.553% of total N, 1.537% of total P, 4.36% of total K. According to statistics, among 600 million tons of rice straw, the nutrient contents of N, P and K are equivalent to more than 3 million tons of urea, more than 700,000 tons of calcium superphosphate and more than 7 million tons of potassium sulfate. Therefore, rice straw returning soil can not only alleviate the imbalance of the proportion of N, P and K in the soil, but also make up for the deficiency of P and K^12^.

Rice straw returning soil affected the content of DOM and the form of heavy metals in soil. Rice straw returning soil will form a large amount of DOM in a short time. With the increase of DOM content, the content of water-soluble heavy metals and organically bound heavy metals in soil will gradually increase^13–15^; DOM may also have the ability to complex heavy metals because it contains a large number of functional groups such as carboxyl, hydroxyl and carbonyl groups. It affects the dissolution of soil REEs through complexation/chelation^4,5,16^. It is reported that rice straw returning soil can reduce the content of exchangeable Zn, Pb and Cu in soil, reduce the activity of heavy metals in soil and reduce the absorption of heavy metals by crops^17–21^. Rice straw returning soil can improve the adsorption of Cu in red soil and reduce the bio-availability of chromium and nickel, but it has no significant effect on the activity of Zn^22,23^. Shan et al. believed that after adding rice straw to the soil, the dissolution of soil Cu and Cd increased due to the release of soluble organic matter in a short time, so as to promote the absorption of Cu and Cd by wheat. As can be seen that the effect of rice straw returning on heavy metals of soil is very complex, and REEs have the general properties of heavy metals^24^. Jin et al. showed that rice straw returning soil affects the concentration of dissolved REEs in soil and the content of REEs in plants, but the mechanism is very complex^2,3^.

Rice straw returning soil significantly affected rice yield, but the mechanism was also complex. Peng et al. believed that rice straw returning soil mainly affected the yield by affecting the number of effective panicles, and the effect of rice straw returning soil combined with nitrogen fertilizer on the yield of late rice was significantly better than that of early rice^25^. Because the effect of rice straw returning soil on the growth of rice roots is pre-inhibition and post-promotion, the growth of single stem and root system is inhibited in the peak tillering stage^26^. Jin and other researchers have shown that different rice straw returning methods have different effects on rice yield in rare earth mining areas. When early rice is planted in the mining area, the growth of rice will be inhibited if rice straw is directly returned to soil, and the dry weight of rice grain, shoot and roots will be significantly lower than that of the control. If rice straw ash is returned to the field in light rare earth mining areas, the dry weight of rice grain, shoot and roots will be significantly increased, and the content of REEs will be reduced, while in heavy REE mining areas, it had little effect on rice growth, root, shoots, grain dry weight and REE content^4,5,15^.

Rice straw returning soil has significant impact on microbial biomass, community structure, diversity and function in paddy soil^27^. Rice straw returning affected the community structure and function of soil bacteria. The research shows that rice straw returning soil combined with chemical fertilizer can significantly increase the number of nitrogen transformation functional bacteria such as soil ammonia oxidizing bacteria, nitrogen fixing bacteria, nitrifying bacteria, denitrifying bacteria and fiber decomposing bacteria^28^. However, some studies have shown that rice straw returning soil will reduce the accumulation of soil organic matter and microbial diversity^29^. With the continuous decomposition of rice straw, the amount of nutrients available to soil microorganisms will decrease, which will further inhibit the activity of soil microorganisms. Rice straw returning soil may lead to the decrease of nifH gene expression level and nitrogen fixation activity^28^. Jin et al. showed that when planting early rice in rare earth mining areas, adding rice straw to soil will cause α-diversity of soil bacteria to decrease and reduce the abundance of beneficial bacteria of *Acidobacteria* and *Nitrospirae,* but promote the growth of *pseudorhodoferax*, *phenobacterium* and other bacteria^4,5^.

Rice straw returning affects the abundance and diversity of endophytic bacteria in plants, thus affecting plant growth indicators and anti biotic systems. Microorganisms in plants are one of the key factors affecting plant growth, nutrition, and health. They help plants obtain nutrients, inhibit plant pathogens, and resist biological and abiotic stresses^30^. When *Streptomyces albidoflavus Osilf 2* was inoculated in conditions of greenhouse and field, the rice disease index decreased by 18.0% and 19.6%, respectively, indicating that Osilf 2 has significant biocontrol activity and host defense stimulation ability^31^. The antagonistic ability of *Streptomyces sporocinereus osish-2* against *Magnaporthe grisea* is related to the competition of Fe^32^. Rice endophytic bacteria*-Streptomyces hygroscopicus osish-2* has obvious antagonistic activity against *Magnaporthe oryzae*. *Endophytic Streptomyces osish-2* has the potential as a biocontrol agent of rice blast. The effect of rice endophytic bacterium *Burkholderia cepacia* on the growth of rice plants under the condition of a greenhouse was studied, and result showed that the absorption of nitrogen, phosphorus, and potassium by rice inoculated with bacterial endophytic bacteria increased significantly, which may be due to the promotion of root growth through indoleacetic acid (IAA) to absorb more soil nutrients^33^. Rice endophytic bacteria can promote the formation of auxin, stimulate plant development, and promote the growth of rice plants and rice yield. Leguminous rhizobia significantly increased the growth of shoots and roots of hybrid rice and improved the grain yield and nitrogen utilization rate of agricultural fertilizers^34^. In the pot experiment on the alleviation of Cd stress in rice, maltophilia R5-5 exhibited the greatest potential to reduce the Cd contents of rice roots and leaves, which were reduced by 81.33% and 77.78%, respectively. Inoculation with maltophilia R5-5 can alleviate heavy metal pollution in rice fields^35^.

REEs not only have the general properties of heavy metals, but also have their own characteristics. Zhang et al*.* showed that rice straw returning soil affected the form of soil rare earth elements and the physical and chemical properties of the soil, affected the rhizosphere and phyllosphere bacteria of rice, and the community structure of bacteria in rice roots and leaves, thus affected the growth promoting function of rice endophytic bacteria and rice growth, but the mechanism was very complex^30^. It is not clear how endophytic bacteria affect the REE content of rice in the mining area.

At present, the study on the effect of rice straw returning soil on endophyte of plants in rare earth polluted habitats has not been found. What is the relationship between the influence of rice straw returning on soil–plant ecological effects, especially on endophytic bacteria, and the amount of rice straw returning, the way of rice straw returning, the nature of soil and other factors? The paddy soil of typical ionic rare earth mining area is taken as the research object, this paper discusses the effects of different rice straw returning amounts and fertilization methods on bacteria in rice rhizosphere, root, leaf and phyllosphere, so as to provide theoretical and practical guidance for rice planting and rice straw returning soil scientifically in mining area.

## Materials and methods

### Soil collection and material preparation

Sampling points are arranged at about 1000 m (the longitude and latitude is 115° 42′ 8″ E, 24° 52′ 55″ N) in the rare earth mining area of Shipai village, Wenfeng Township, Xunwu County, South Jiangxi Province and its surrounding areas. Each point was distributed in a snake shape, and 200 kg of 20 cm soil from the surface was collected. The soil sample was air dried naturally and screened through 100-mesh sieve for standby. The test soil was loaded into a black PVC bucket with a height of 25 cm and a diameter of 21 cm, and the barrel was filled with 5 kg of soil. This experiment mainly investigated the effects of 1% and 2% rice straw returning amounts, and different combinations of N, P, and K fertilizers on rice soil ecology, especially rice endophytes. 1% of rice straw returning soil is equivalent to half of the rice straw returning soil, and 2% of the rice straw returning soil is equivalent to all the rice straw returning soil. Half of rice straw returning soil and all of the rice straw returning soil are the two main ways for farmers in the mining area to return rice straw to soil. Because this experiment mainly investigates the impact of rice straw returning soil on P fertilizer and K fertilizer, the combination of chemical fertilizer is divided as NPK, NP, and NK, and 1% and 2% rice straw are added to each. The experimental samples are 0% NPK (CK, i.e. NPK fertilizer applied without rice straw), 1% NPK, 2% NPK, 1% NP, 2% NP, 1% NK, and 2% NK. Each sample had three replicates. To simplify the representation, they were recorded as C, 1% N, 2% N, 1% P, 2% P, 1% K, and 2% K respectively. The composition of each type of fertilizer was: 5.4 g of urea (46.6%N), 0.6 g of potassium chloride (62.9% K_2_O), and 2.0 g of calcium magnesium phosphate 14.0% P_2_O_5_). According to the requirements of the experiment, the required chemical fertilizer and the corresponding amount of rice straw were added and mixed evenly with the soil; rice was planted after 6 days of submergence.

### Potted rice and sample collection

The rice variety, Jiangzao 361, was from Jiangxi Keyuan Seed Industry Co., Ltd, China. Rice seeds of the same size were selected, disinfected, and germinated with 15% H_2_O_2_ solution and then sown into the soil without rare earth pollution for seedling raising. After 4 weeks of growth, the rice seedlings were transplanted into the PVC barrel with prepared culture soil. Two seedlings were transplanted to each barrel. The pot experiment was carried out in the greenhouse. The daily illumination, light duration, temperature, and humidity in the greenhouse were adjusted close to those of the outdoor. The rice was managed according to the growth characteristics of Jiangzao 361. The rhizosphere soil, rice roots, and shoots were collected at the tillering stage while the rhizosphere soil and rice roots were collected at maturity and placed in the refrigerator at − 80 °C, it was used to do molecular experiments. At maturity, the rice roots, shoots, and millet were harvested, washed with deionized water, and weighed after natural air-drying. The millet was dehusked and the unpolished rice was collected. Afterward, the unpolished rice, shoots, and roots were ground into powder and stored them in dry conditions for analysis.

### Chemical analysis

The physical and chemical properties of the soil and the content of nutrient elements were determined at the Nanjing Institute of Science, Ministry of Environment, China. The detection methods were as documented by Zhang et al. The determination of soil pH was carried out according to the agricultural industry standard of the People’s Republic of China NY/T 1121.2-2006. The ratio of soil to solution was 1:2.5, and the determination of soil total phosphorus is Alkali Soluble Molybdenum Antimony Anti Spectrophotometry, which is recommended by the National Environmental Protection Standard of the People’s Republic of China HJ 632-2011. The determination of soil available phosphorus is Sodium Bicarbonate Extraction Molybdenum Antimony Resistance Spectrophotometry, which is recommended by the National Environmental Protection Standard of the People’s Republic of China (HJ 704-2014). and the National Environmental Protection Standard of the People’s Republic of China, HJ 634-2012 of Potassium Chloride Solution Extraction Spectrophotometry, was chosen for the determination of soil ammonia nitrogen, nitrite nitrogen, and nitrate nitrogen. the soil available potassium and slow available potassium was determined using the Agricultural Industry Standard of the People’s Republic of China, NY/T889-2004 and extracted with neutral 1 mol/L ammonium acetate solution, and the content of soil organic matter was determined using the Agricultural Industry Standard of the People’s Republic of China, NY/T 1121.6-2006:The excess potassium dichromate sulfuric acid solution was used to oxidize soil organic carbon and the excess potassium dichromate was titrated with ferrous sulfate standard solution under the conditions of heat, and the amount of organic carbon is calculated from the amount of potassium dichromate consumed according to the oxidation correction coefficient, and then multiplied by the constant 1.724, which is the content of soil organic matter. The test results can be seen in Table 1.

**Table 1:** Soil physical and chemical properties and nutrient elements (mg/kg).

Sample name	Total N	Ammonia N	NOx-N	Total P	Available P	Rapidly available K	Organic matter (%)
C-1	2624.05	311.43	34.27	1141.23	87.10	81.50	5.67
N1-1	203,450	178.00	29.82	331.29	13.72	81.50	3.93
N2-1	2063.64	491.84	33.42	377.69	12.77	642.02	4.19
P1-1	1587.52	198.86	30.82	431.15	17.20	76.31	3.87
P2-1	1742.92	252.75	30.70	319.30	18.84	401.19	3.99
K1-1	1849.89	154.94	31.07	249.98	11.40	78.99	3.85
K2-1	1822.85	246.01	31.39	344.04	15.16	418.74	4.56
C-2	2839.02	113.75	31.65	1202.01	106.47	111.49	5.33
N1-2	1709.53	71.92	28.73	193.84	7.10	84.32	3.34
N2-2	1642.81	49.72	29.91	224.06	7.65	117.99	3.47
P1-2	1765.76	143.33	29.14	213.86	9.08	115.65	3.33
P2-2	1543.42	110.72	27.45	155.21	9.63	132.62	3.59
K1-2	1265.60	66.68	28.500	214.02	6.22	90.32	3.38
K2-2	1675.43	61.35	28.61	243.78	5.06	86.16	3.57

Soil sample (0.2 g) was weighed into a quartz glass tube (three replicates for each sample) and 5 mL aqua regia (HNO_3_:HCl = 3:1) was added. It was soaked overnight and digested in an open digester. The digested sample was placed in the fume hood to volatilize the acid and then transferred into a 50 mL constant volume tube. It was later diluted to 50 mL with ultrapure water and shaken well, then filtrated by 0.45 µM filter membrane, and the sample was analyzed using ICP-MS. The national standard material, GBW07405, was used for quality control throughout the digestion process. The content of REEs in the soil samples collected from the rare earth mining area was 838.16 mg/kg. Crushed unpolished rice (0.2 g), shoot (0.2 g), and roots (0.1 g) were placed in 50 mL polyethylene centrifuge tubes (three replicates for each sample). Then, 3 mL of high-grade pure nitric acid was added to it. The samples were soaked overnight and digested in microwave digestion oven (Mars, Matthew Inc., USA), Digestion procedure: first, the samples were heated to 120 °C for 5 min, after which the temperature was raised to 160 °C for 15 min. The shrub branches and leaves of the national first-class reference material, GBW07603 (GSV-2), (Institute of Geophysical and Geochemical Exploration, Ministry of Geology and Mineral Resources of China) were used for quality control in the entire digestion process. The digestion procedures of plants and soil were as reported by Zhang et al. The digestion solution was fixed to 40 mL with ultrapure water. After filtration by 0.45 μM filter membrane, the contents of 15 REEs (Y, La, Ce, Pr, Nd, Sm, Eu, Gd, Tb, Dy, Ho, Er, Tm, Yb and Lu) were determined by ICPMS-2030.

### Pretreatment of soil and plant samples and DNA extraction

The loose and massive soil around the rice root samples was removed. The rice root was immersed in 15 mL PBS (0.1% Tween 80) solution and shaken over a shaking table for 10 min; the resulting suspension was introduced into a sterile centrifuge tube. This process was repeated three times. After mixing the suspension obtained each time, the final suspension was centrifuged at rotating speed of 6000×*g* for 5 min. The precipitate particles obtained were rhizosphere soil samples and were stored at − 80 °C for subsequent DNA extraction. The rice roots shaken over the shaking table were washed with 75% ethanol for 10 min, 2.5% sodium hypochlorite for 10 min, and sterile water for 5 times. They were ground in PBS with a sterile mortar and pestle, added to a centrifuge tube and washed, allowed to stand for 30 min, and then centrifuged at rotating speed of 6000×*g* for 5 min; the cell particles (endophytic bacteria samples) thus obtained were stored at − 80 °C for subsequent DNA extraction^36,37^. The leaves were put into a 250 ml sterile Erlenmeyer flask, stirred vigorously in 100 mL PBS (0.1% Tween 80) for 30 min, and then put in an ultrasonic cleaning water bath for 10 min. The supernatant (sample of rhizosphere bacteria) was concentrated at 0.22 μM on the nitrocellulose membrane filter. Before DNA extraction, the membrane was stored at − 80 °C. The leaves were washed in 75% ethanol for 3 min, 2.5% sodium hypochlorite for 5 min, and sterile water five times. The leaves were ground in PBS with a sterile mortar and pestle. Added to a centrifuge tube and washed, and then allowed to stand for 30 min. After mixing the suspensions obtained each time, centrifuge them at the speed of 6000×*g* for 5 min The extracted cell microspheres (endophyte samples) were stored at − 80 °C before DNA extraction. The DNA of rhizosphere soil and bacteria samples in the roots, leaves, and phyllosphere was extracted by FastDNA^TM^SPIN Kit for soil (MP Biomedicals LLC, USA). DNA concentration and purity were measured using the micro ultraviolet–visible spectrophotometer, NanoDrop2000, and then stored in a refrigerator at − 20 °C for subsequent 16S rRNA gene analysis. Experimental methods were according to Zhang et al.^30^.

### Amplification and sequencing of 16S r RNA gene

The specific process is as follows: DNA (about 10 ng) was used as a template and primers with barcodes were used to amplify specific regions. Bacterial 16S rRNA V4 region was amplified with the primer pair 515F (5ʹ–GTGCCAGCMGCCGCGGTAA–3ʹ). The amplification reaction system was 25 μL, including 12.5 μL of 2× Taq MasterMix (TaKaRa, Dalian), 1 μL of pre-primer and 1 μL of post-primer, 9.5 μL of sterilized ultrapure water, and 1 μL of DNA template. The amplification conditions were: 94 °C for 3 min, (94 °C for 30 s, 56 °C for 60 s, 72 °C for 60 s) × 30 cycles, 72 °C for 10 min. The obtained PCR product was detected using 1% agarose gel electrophoresis. The target strip was recovered using a gel Recovery Kit (DNA Gel Extraction Kit), and the concentration and quality of the recovered DNA samples were determined by Nanodrop. The library was constructed using TruSeq® DNA PCR-Free Sample Preparation Kit. The constructed library was quantified with qPCR, and the quantified library was sequenced. Sequencing samples included soil and plant samples at the tillering and maturity stages (rhizosphere soil samples, phyllosphere bacteria samples, leaf endophytic bacteria samples, and root endophytic bacteria samples). The data obtained after sequencing were controlled with the FastQC software. Barcodes were cut using extract_barcodes.py, primers were cut with the Cutadapt software, and the Usearch10.0 software was used to remove redundancy and chimerism. Sample data that were too small were removed and UNITE database was used for species annotation. The experimental method was according to Zhang et al.^30^.

### Statistical analysis

The species diversity involved in this paper are α-diversity and β-diversity, and α-diversity includes richness, evenness, Inv_Simpson index, etc. The analyses were implemented through the “vegan” package. The microbial community structure was analyzed using ordination analysis, which was divided into two types, non-binding analysis (microbial community analysis) and binding analysis (relationship between microbial community and environmental factors). The non constraint analysis method involved in this paper is non metric multidimensional scaling (NMDS).

We use analysis of variance (ANOVA) and analysis of similarities (ANOSIM) in this paper to analyze the significant differences between the mean values of two or more samples. Pearson test, canonical correspondence analysis (CCA), and variation partition analysis (VPA) based on CCA were used to analyze the contribution of environmental variables to the bacterial community. Origin9.0 and R software are used for drawing, and Metasee software and Qiime software are used for data visualization.

### Ethics statement

Statement on guidelines as experimental research and field studies on rice. Experimental research and field studies on rice (cultivated rice and wild rice), including the collection of rice comply with relevant institutional, national, and international guidelines and legislation, and this studies comply with local and national regulations. The measurement process of microorganism and rare earth content in different parts of rice will not affect the local soil microorganism and ecological environment, etc. During the process of experiment, aseptic sampling was carried out to avoid contamination, and the research was evaluated and agreed by the environmental protection authorities of the local government.

## Results

### Effects of rice straw returning soil on soil physical and chemical properties, rice biomass and REE content

Table 1 shows that the total nitrogen accumulated in the soil after rice straw returning soil decreased in both the tillering and maturity stages. At the tillering stage, the total nitrogen in P1 and P2 decreased the most, followed by that in K1 and K2, and then in N1 and N2; at the maturity stage, the decrease of soil total nitrogen from more to less was in the order: K1, P2, N2, K2, N1, and P1. Except for P2 soil at the tillering stage, the soil ammonia nitrogen after rice straw returning soil decreased at both the tillering and maturity stages. At the tillering stage, no matter the fertilization method adopted, the ammonia nitrogen of 1% rice straw returning soil was significantly lower than that of 2% soil, while at the maturity stage, the ammonia nitrogen of 2% rice straw returning soil was lower than that of 1% soil. No matter in tillering stage or maturity stage, the soil nitrate nitrogen of rice straw returning decreased, but it was not significant. Whether in tillering stage or maturity stage, rice straw returning soil increased the content of total P of soil.

At the tillering stage, the increase from high to low was in the order: P1, N2, K2, N1, P2, and K1; at the maturity stage, the increase from high to low was in the order: K2, N2, N1, K1, P1, and P2. At the tillering stage, rice straw returning significantly increased the content of soil available P, and the increase from high to low was in the order: P1, P2, K2, N1, N2, and K1. At the maturity stage, the content decreased, and the decrease from high to low was K2, K1, N1, N2, P1, P2. At the tillering stage, 2% rice straw returning significantly increased the content of soil available potassium, and the increase from high to low was as follows: N2, K2, P2. The effect was more complex at maturity. The soil organic matter increased at both the tillering and maturity stages with rice straw returning, and the content of soil organic matter with 2% rice straw returning was higher than that with 1% rice straw returning.

**Figure 1:**
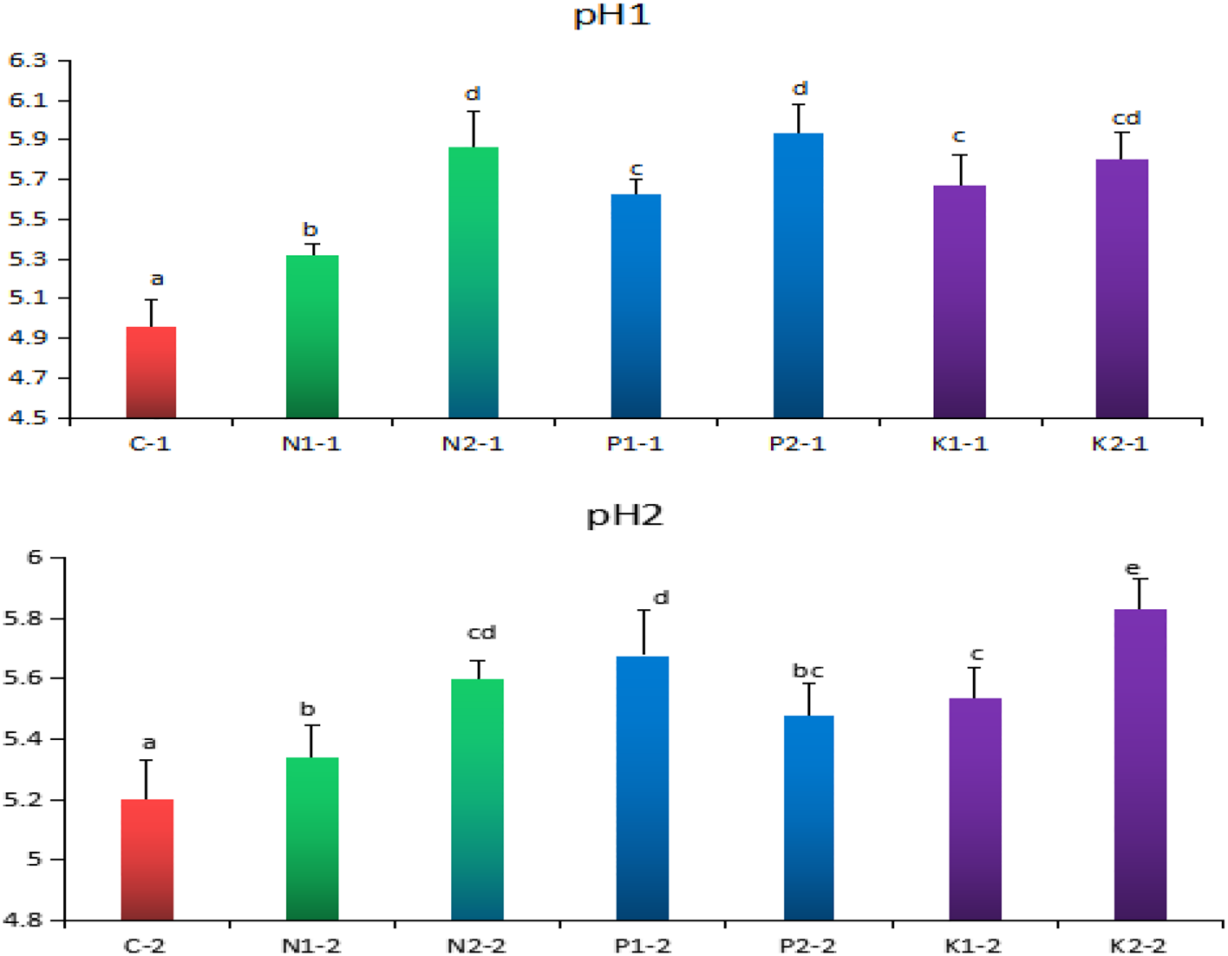
Effect of rice straw returning soil on soil pH value. Different letters indicate significant differences between potted rice in different soils, “ − 1” indicates tillering stage and “ − 2” indicates maturity stage, the same below.

Figure 1 shows that rice straw returning at the tillering and mature stages improves the soil pH value. The increase of soil pH of 2% rice straw returning soil in tillering stage was higher than that of 1% rice straw returning soil, and the pH of P2, N2 and K2 was 16.4%, 15.3%, and 14.5% higher than that of C respectively; At the maturity stage, pH of K2, P1, and N2 increased by 10.8%, 8.40%, and 7.1% respectively compared with C.

**Figure 2:**
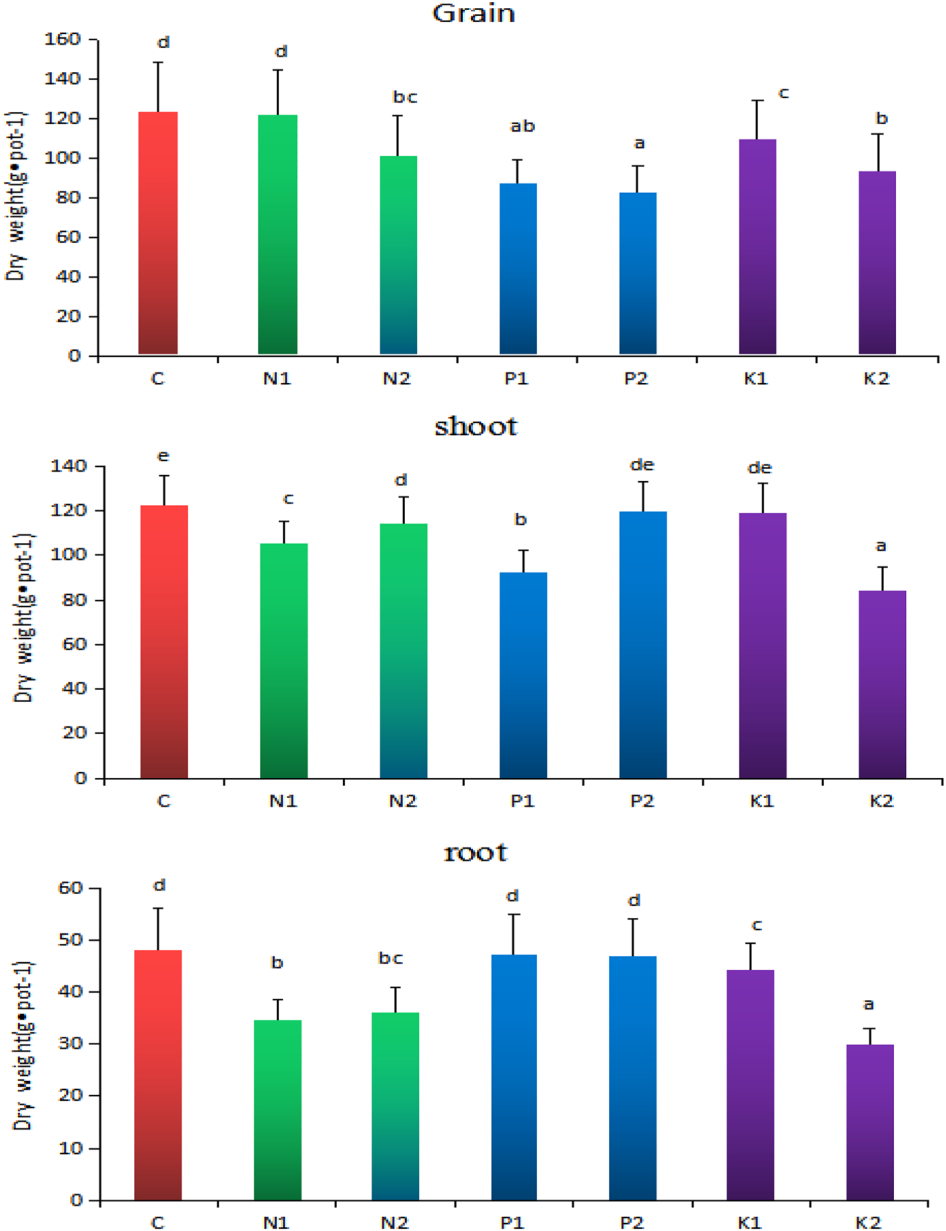
Effects of different rice straw returning methods on dry weight of rice roots, shoots and grains. Different letters indicate significant differences between potted rice in different soils.

Figure 2 shows that the dry weights of the roots, shoots, and grains of rice (C) without adding rice straw and with 1% rice straw combined with NPK fertilizer (N1) are higher than those of 2% rice straw combined with NPK fertilizer (N2), 1% and 2% rice straw combined with NP fertilizer (P1 and P2), and 1% and 2% rice straw is combined with NK fertilizer (K1 and K2). The dry weight of the rice roots, shoots, and grains was as: N1 > N2, P1 > P2, K1 > K2, N1 > P1 > K1, N2 > P2 > K2.

**Figure 3:**
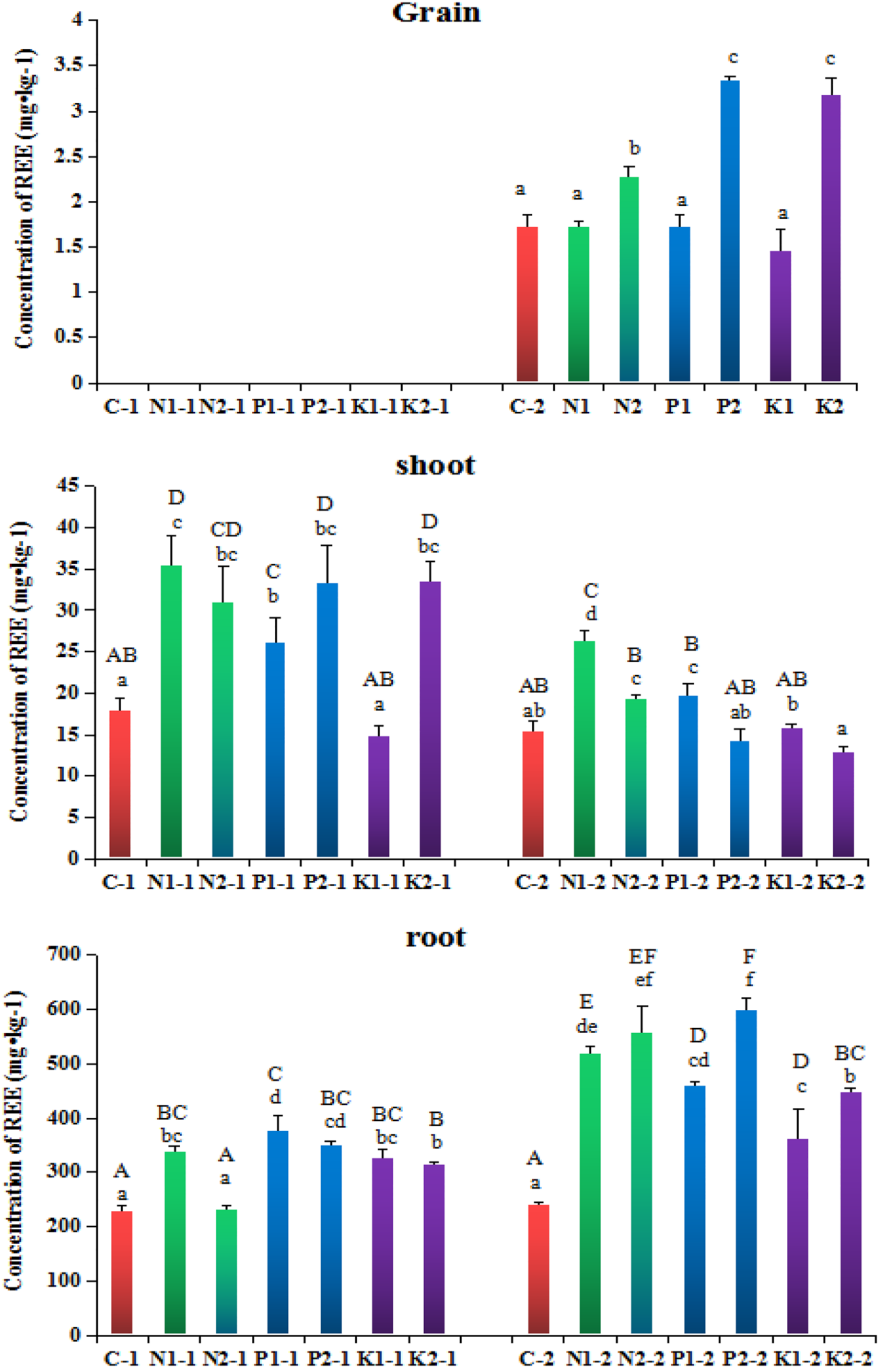
Effects of different rice straw returning methods on the content of REEs in rice roots, shoots and grains. Different letters indicate significant differences between potted rice in different soils (lowercase letters indicate the significant difference within the same stage and uppercase letters indicate the significant difference between the two stages), P < 0.05.

Figure 3 shows that different rice straw returning amount and different chemical fertilizer application at both the tillering and mature stages improved the REE content of rice roots, and contents of N1, N2, P1, P2, K1, and K2 at the tillering stage increased by 32.5%, 0.6%, 39.0%, 34.8%, 30.0%, and 27.5% respectively, and the increases in N1, N2, P1, P2, K1 and, K2 at the mature stage was more significant, namely 53.7%, 57.0%, 47.7%, 59.8%, 33.6%, and 46.4% respectively. Rice straw returning increased the content of REEs in the shoots of most rice samples, except for K1 samples, the content of REEs in the shoots of N1, N2, P1, P2, and K2 increased by 49.3%, 42.0%, 31.2, 46.1%, and 46.5% respectively at the tillering stage. At the maturity stage, except for P2 and K2, the content of REEs in the shoots of N1, N2, P1, and K1 increased by 41.3%, 20.7%, 21.9%, and 2.4% respectively. Rice straw returning significantly improved the content of REEs in N2, P1, and K2 of rice grains by 24.5%, 48.5%, and 45.9%, respectively. The effects in N1, P2, and K1 were not significant.

**Figure 4:**
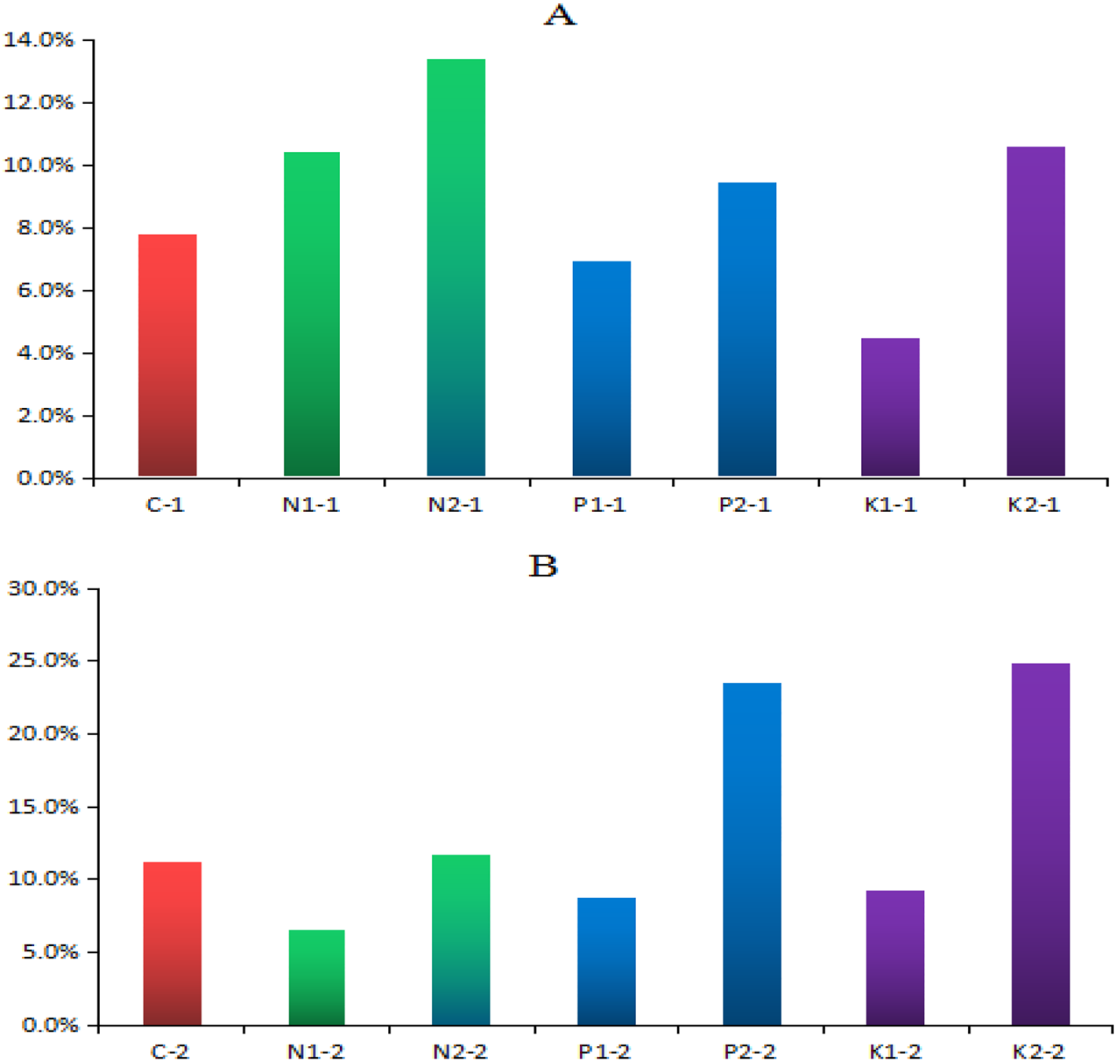
Mobility of REEs from root to shoots and shoot to grain. (**A**) Shows the migration rate of REEs from root to shoot at tillering stage, and (**B**) shows the migration rate of REEs from shoot to grain at maturity.

Figure 4A shows that rice straw returning affects the mobility of REEs from root to shoot at the tillering stage Under the same fertilization conditions, the mobility of REEs in 2% rice straw returning soil was higher than that in 1% soil, which in turn was higher than that in soil without adding rice straw. Compared with C, mobility in N2, P2, and K2 increased by 41.7%, 17.3%, and 26.2% respectively. Figure 4B shows that rice straw returning affects the migration rate of REEs from shoots to grains at maturity stage. Under the same fertilization condition, the migration rate in 2% rice straw returning soil was higher than that in 1%, which in turn was higher than that in soil without adding rice straw. Compared with C, mobility in N2, P2, and K2 increased by 4.8%, 52.5%, and 54.9%, respectively, and that in N2 increased by 43.9%, 62.8%, and 62.8% respectively compared with N1, P2–P1, and K2–K1.

### Effects of rice straw returning soil on the bacteria diversity of root, rhizosphere, leaf and phyllosphere of rice

**Figure 5:**
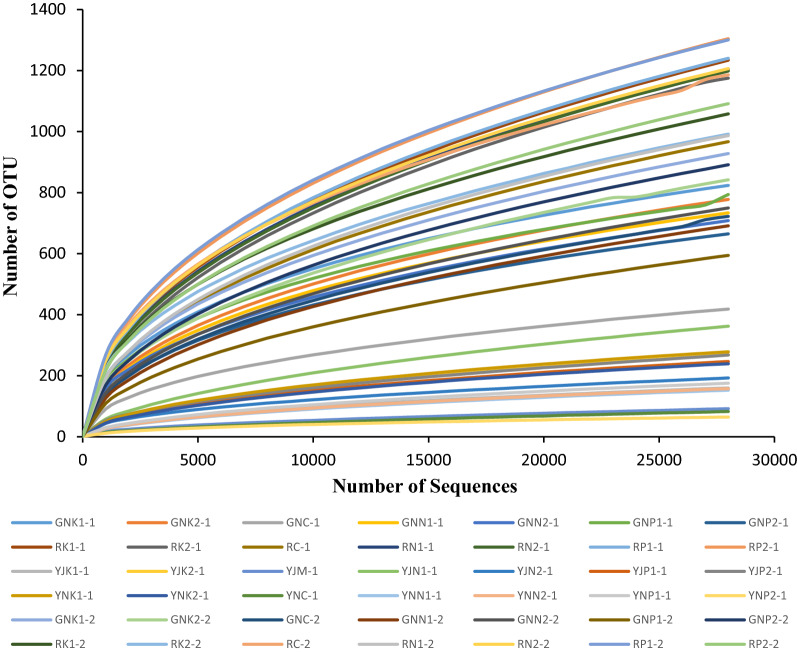
Dilution curve of bacterial 16S gene sequence. (“GN” indicates bacteria in roots, “R” indicates bacteria in rhizosphere, “YJ” indicates bacteria in phyllosphere, “YN” indicates bacteria in leaf, the same below).

REEs in soil affect α-diversity of bacteria, including abundance index: OTU number, Chao; Diversity index: Shannon, inv Simpson, richness; Evenness index: evenness. Under the condition of extracting the same sequence, the more the number of OTUs, the higher the species richness. Figure 5 shows that the species richness of bacteria in rhizosphere > bacteria in root > bacteria in phyllosphere > bacteria in leaf. The species richness of rhizosphere bacteria of P1, P2, K1, and N1 at the tillering stage and those of P1 and N2 at the maturity stage was highest. The species richness of bacteria in the root of K1, K2 at the tillering stage and the maturity stage and P2 at the maturity stage was the highest. Furthermore, the species richness of bacteria in the rhizosphere of N1 was the highest, while for bacteria in the leaf, species richness was the highest in K1 and K2 in the maturity stage. In the dilution curve, the curve basically tended to be flat, indicating that the number of samples was reasonable.

**Figure 6:**
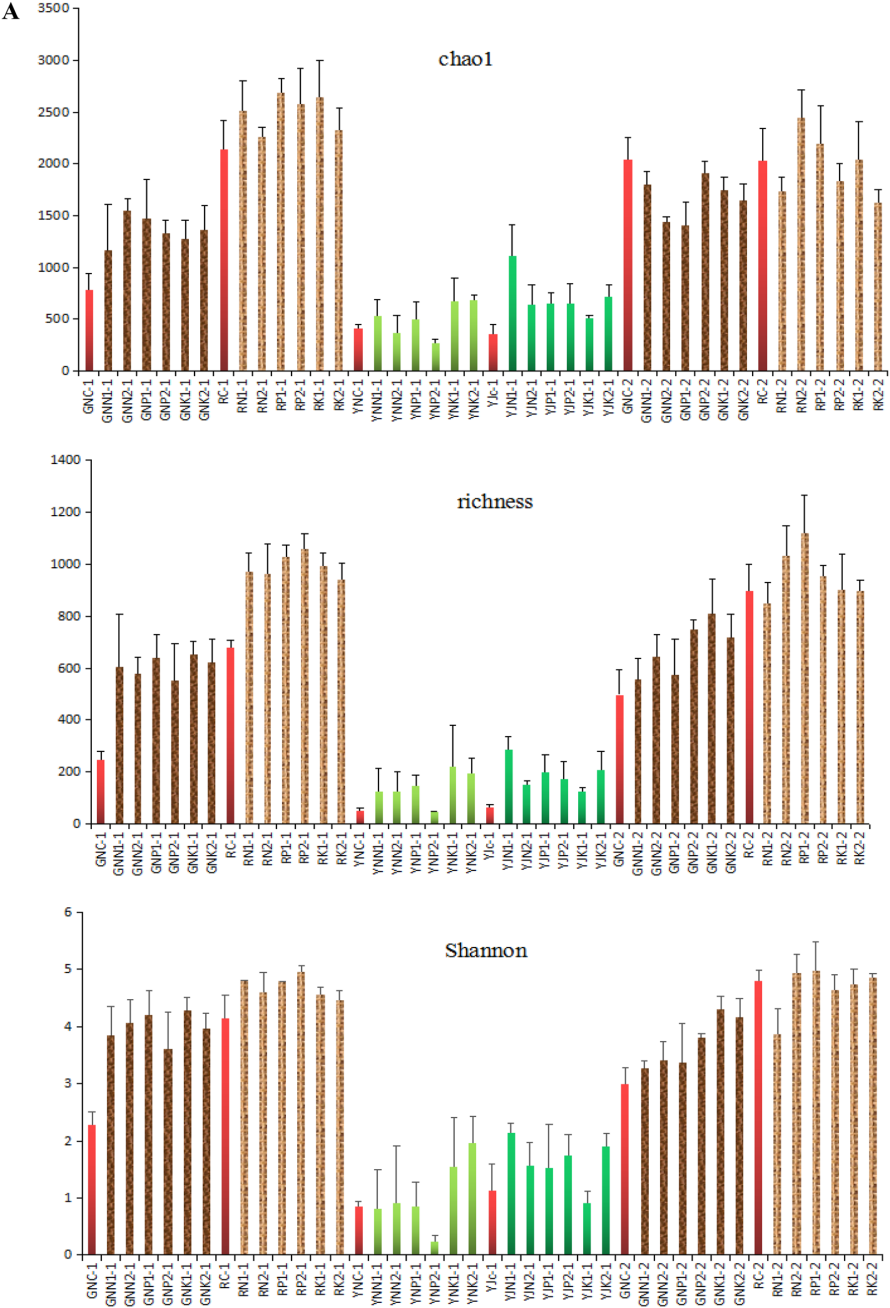

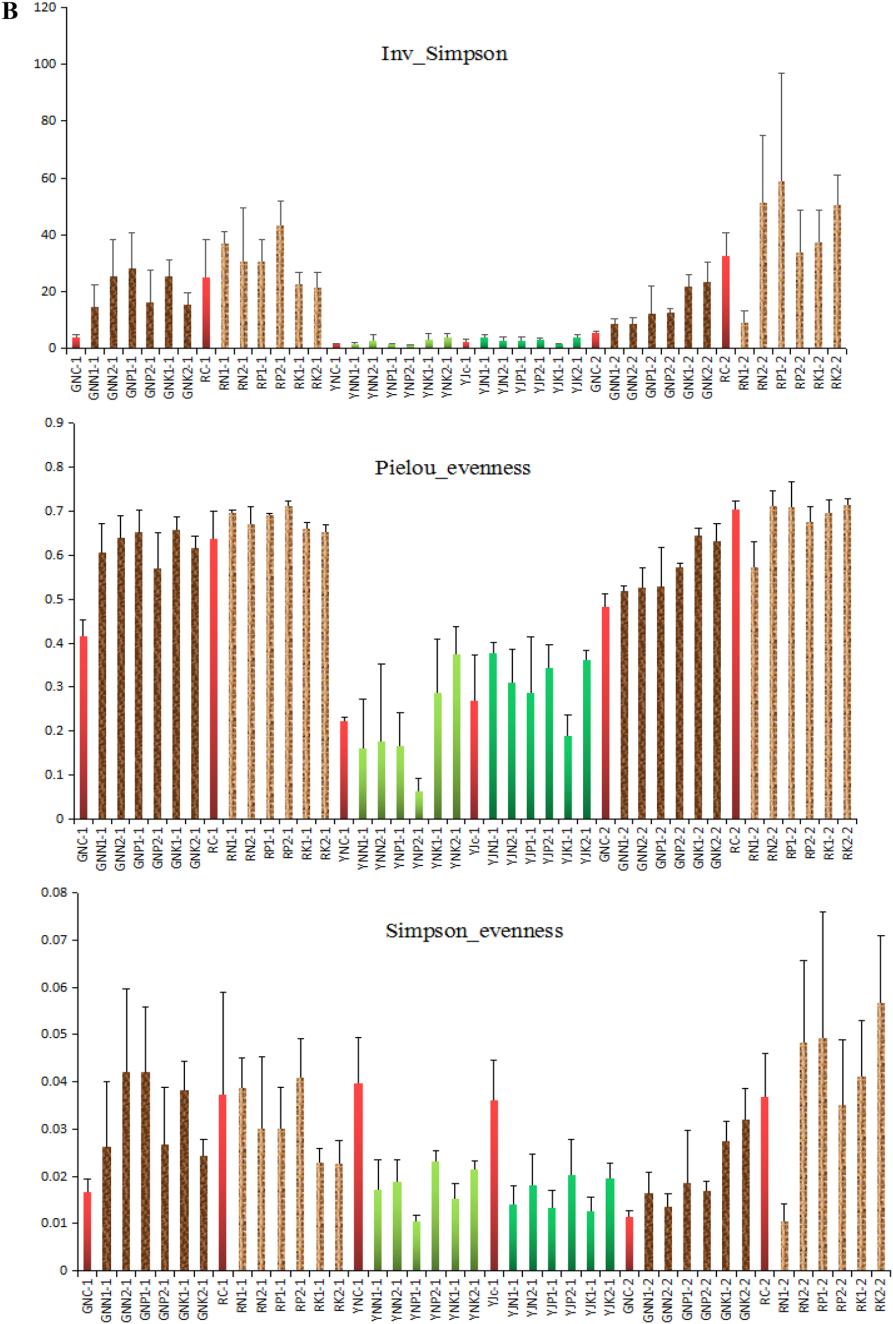
(**a**) α-diversity (chao-1, richness, Shannon) of bacteria in root, rhizosphere, leaf, and phyllosphere of rice at tillering stage and maturity stage. (**b**) α-diversity (Inv_simple, Pielou_evenness, Simpson_evenness) of bacteria in root, rhizosphere, leaf and phyllosphere of rice at tillering stage and maturity stage.

Figure 6a shows that the different amounts of rice straw returning have different effects on the Chao1 index of bacteria in rice roots, rhizosphere, leaves, and phyllosphere. At the tillering stage, different rice straw returning amounts and fertilization conditions significantly improved the Chao1 index of bacteria in the root, with an increase of 47.9–97.2%. The growth range of rhizosphere bacteria ranged from 5.6 to 25.4%, which was less than that of bacteria in the roots. At the tillering stage, the effects of different rice straw returning amounts and fertilization conditions on the Chao1 index of bacteria in leaves were complex, while they significantly increased the Chao1 index of bacteria in phyllosphere, with an increase of 40.8–206.4%. At the maturity stage, different amounts of rice straw returning reduced the Chao1 index of bacteria in rice root, with a decrease range of 6.1–30.8%. The effects on rhizosphere bacteria were inconsistent as some increased while others decreased.

Figure 6a shows that different rice straw returning amounts significantly improved the richness index of bacteria in the rhizosphere and root of rice at the tillering stage. Compared with GNC-1, the richness index of root bacteria in soil with rice straw increased by 125.1–166.8%; compared with RC-1, the richness index of rhizosphere bacteria in soil with rice straw increased by 38.3–56.0%. Except for YP2-1, the richness index of bacteria in leaves increased by 149.3–344.6%, and that in the rice phyllosphere increased by 98.4–359.0% in soil with the addition of rice straw. At maturity, the addition of rice straw increased the richness index of bacteria in the root of rice, with an increase of 11.4–62.3%. Rice straw returning had no significant effect on the richness index of bacteria in the rhizosphere of rice.

As can be seen in Fig. 6b, the addition of rice straw at the tillering stage significantly increased the inv_Simpson index of the bacteria in the rice roots by 261.1–586.4%. Besides the increase in RK1-1 and RK21-1, the addition of rice straw also improved the inv_Simpson index of bacteria in the rhizosphere of rice, with an increase of 23.0–73.1%. On the other hand, the effect of rice straw addition on the inv_Simpson index of leaf bacteria was complex, and the index varied greatly with different treatments. Except for YJK1-1, the addition of rice straw improved the inv_Simpson index of bacteria in the rice rhizosphere, with an increase of 16.7–69.8%; at maturity, the addition of rice straw significantly improved the Inv_Simpson of bacteria in rice roots, with an increase of 56.9–318.3%. Except YJN1-1, the addition of rice straw improved inv_Simpson index of bacteria in rhizosphere of rice, with an increase of 3.7–80.1%.

Figure 6b shows that the addition of rice straw at the tillering stage significantly improved Pielou_evenness of bacteria in the rice roots, with an increase of 37.8–58.9%. The effect of the addition of rice straw on the Pielou_evenness of bacteria in the rhizosphere of rice was not significant. The effects of the addition of rice straw on the Pielou_evenness of bacteria in rice leaves was complex. The index of samples YNK1-1 and YNK2-1 increased, while that of other samples decreased. Except for YJK1-1, the addition of rice straw improved the Pielou_evenness of bacteria in the phyllosphere of rice, with an increase range of 6.6–40.4%. At the maturity stage, the addition of rice straw improved the Pielou_evenness of bacteria in rice root, with a range of 7.4–33.4%. The effect of addition of rice straw on the Pielou_evenness of bacteria in rice rhizosphere was not significant at the maturity stage.

**Figure 7:**
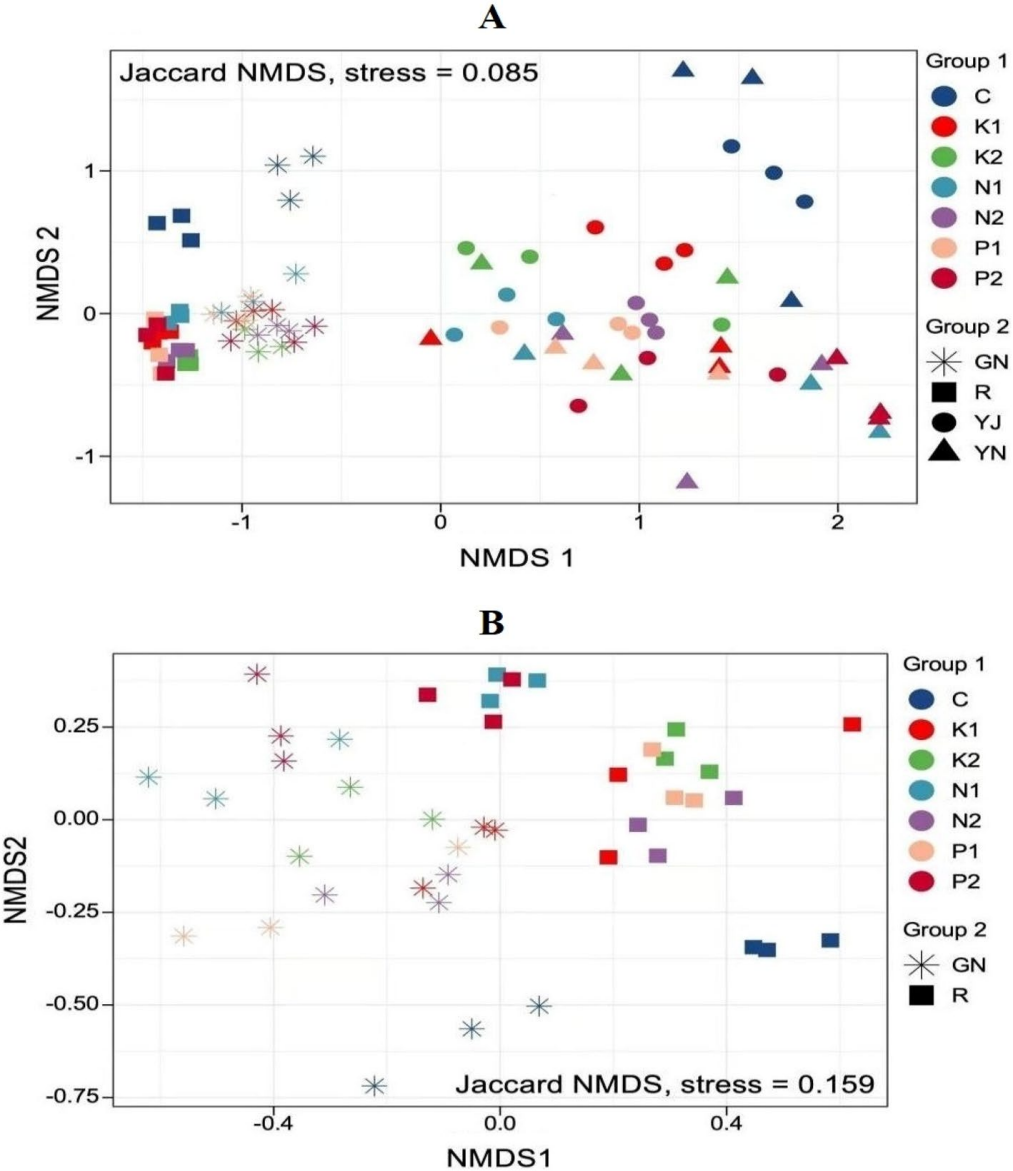
NMDS analysis of β-diversity of bacterial community structure of rice in different ways of rice straw returning (Figure (**A**) shows NMDS analysis of bacterial community structure of rice at tillering stage, Figure (**B**) shows NMDS analysis of bacterial community structure of rice at maturity stage; (**C**) in the figure represent the sample under conditions of no rice straw, that is, the control; K1 and K2 represent 1% and 2% rice straw combined with NK fertilizer; N1 and N2 represent represent 1% and 2% rice straw combined with NPK fertilizer, P1 and P2 represent 1% and 2% rice straw combined with NP fertilizer).

The previous analysis is α-diversity, which is the diversity within the sample. The differences between samples were also analyzed using β-diversity. Nonmetric multi-dimensional scaling (NMDS) is a data analysis method that simplifies the research objects (samples or variables) in multi-dimensional space to low-dimensional space for positioning, analysis, and classification, while retains the original relationship between objects, which can better reflect the nonlinear structure of ecological data. The stress coefficient was used to measure the quality of NMDS results. Figure 7A shows that the stress value obtained from the NMDS analysis of rice samples at tillering stage was 0.09, which is less than 0.1, indicating that non-metric multidimensional scaling NMDS Bray–Curtis is a good fit, and different rice straw returning amounts have significant effect on β-diversity of bacteria. It can be seen from Fig. 7B that the stress values obtained from NMDS analysis of rice samples at maturity stage was 0.159, less than 0.2, indicating that there were significant differences between the samples. Figure 7A,B show that the differences between samples are manifested in many aspects: first, from the vertical level, the differences between samples of rice root and shoot were significant; second, from the horizontal level, there were significant differences between the bacteria in the root and those in the rhizosphere, and between the bacteria in the leaf and those in the phyllosphere; third, returning of rice straw to soil under different conditions resulted in significant differences among the samples.

### Effect of rice straw returning soil on the community structure of bacteria in rhizosphere, root, leaf and phyllosphere of rice

**Figure 8:**
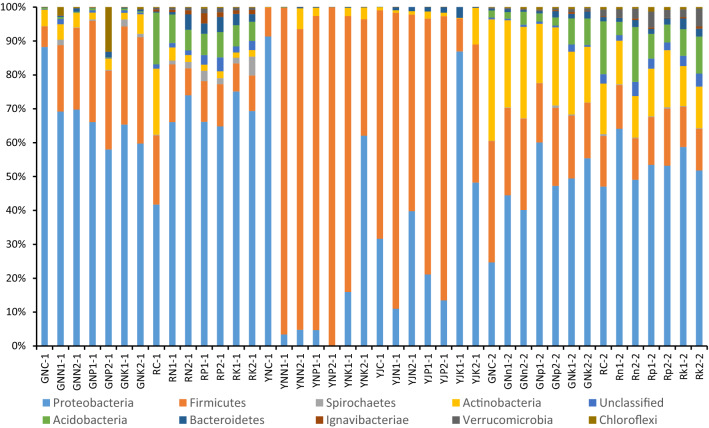
Histogram in Phylum level.

As is seen in Fig. 8, the bacteria mainly include Proteobacteria, Firmicutes, Actinobacteria, Acidobacteria, Chloroflex, and other bacteria. The addition of rice straw significantly reduced the abundance of Proteus in the rice roots and leaves at the tillering stage, and in the rice rhizosphere at maturity by 21.0–34.3%, 32.1–99.7%, and 1.5–47.0%, respectively. As for the reduction range of Proteus in rice root at tillering stage, the effect of addition of 2% rice straw is better than that of addition of 1% rice straw. The abundance of Proteobacteria in the rhizosphere at the tillering stage and in the root at the maturity stage increased by 55.1–78.9% and 53.6–111.2%, respectively; the effects of different rice straw returning conditions on the abundance of Proteobacteria in rice leaves at tillering stage were inconsistent. The abundance of Proteobacteria in the phyllosphere of samples YJN1-1, YJP1-1, and YJP2-1 decreased by 65.2%, 33.4%, and 57.4%, respectively, while the abundance of Proteobacteria in the phyllosphere of samples YJN2-1, YJK1-1, and YJK2-1 increased by 25.7%, 174.6%, and 52.3%, respectively.

The addition of rice straw greatly increased the abundance of Firmicutes in the roots and leaves at the tillering stage by 218.7–411.6% and 303.2–1069.9%, respectively. The abundance of Firmicutes in rice rhizosphere at the tillering stage, and in rice rhizosphere and root at maturity decreased by 16.3–61.1%, 0.5–64.5%, and 42.3–70.3%, respectively; the effects of different rice straw returning conditions on the abundance of Firmicutes in rice phyllosphere at the tillering stage were inconsistent. The abundance of Firmicutes in the phyllosphere of samples YJK1-1 and YJK2-1 decreased by 39.6% and 85.6%, respectively, while the changes in other samples were not significant.

The addition of rice straw decreased the abundance of Actinobacteria in rice roots and rhizosphere at the tillering stage, and in rice roots at the maturity stage by 6.7–60.4%, 80.1–92.2%, and 28.8–61.5% respectively. The effects of different rice straw returning conditions on the abundance of Actinobacteria in the leaves and phyllosphere of rice at the tillering stage were inconsistent: some samples exhibited increase while others exhibited decrease. The abundance of this phylum in the leaves of sample YNP2-1 decreased by 88.3%, and its abundance in the leaves of other samples increased by 11.7–5165.0%. The abundance of Actinobacteria in the rice phyllosphere of YJK1-1 decreased by 79.7%. The addition of rice straw had no significant effect on the abundance of Actinobacteria in the rhizosphere of rice at the maturity stage.

Different rice straw returning conditions significantly reduced the abundance of Acidobacteria in the rhizosphere of the sample, with a reduction range of 45.0–63.3%. Except for sample RN2-2, the abundance of Acidobacteria and Rhizosphere decreased significantly, with a decrease range of 30.3–72.8% at the maturity stage. Different amounts of rice straw had different effects on the abundance of Acidobacteria in rice roots at the tillering stage. The abundance of Acidobacteria in rice roots under 1% rice straw condition increased by 26.8–38.0% and that in rice roots under 2% rice straw condition decreased by 13.4–62.5%. Irrespective of addition of rice straw, the abundance of Acidobacteria in the leaves and phyllosphere of the samples was very low. At the maturity stage, the abundance of Acidobacteria in the roots of GNN2-2, GNK1-2, and GNK2-2 samples increased by 72.7%, 212.3%, and 213.1% respectively, while the increase in other samples was not significant. Except for sample RN2-2, the abundance of Acidobacteria in rhizosphere decreased significantly, with a decrease range of 30.3–72.8%. The addition of rice straw greatly increased the abundance of Spirochaetes, Bacteroidetes, Ignavibacteria, Verrucomicrobia, and Chloroflexi in the roots, rhizosphere, leaves, and phyllosphere.

**Figure 9:**
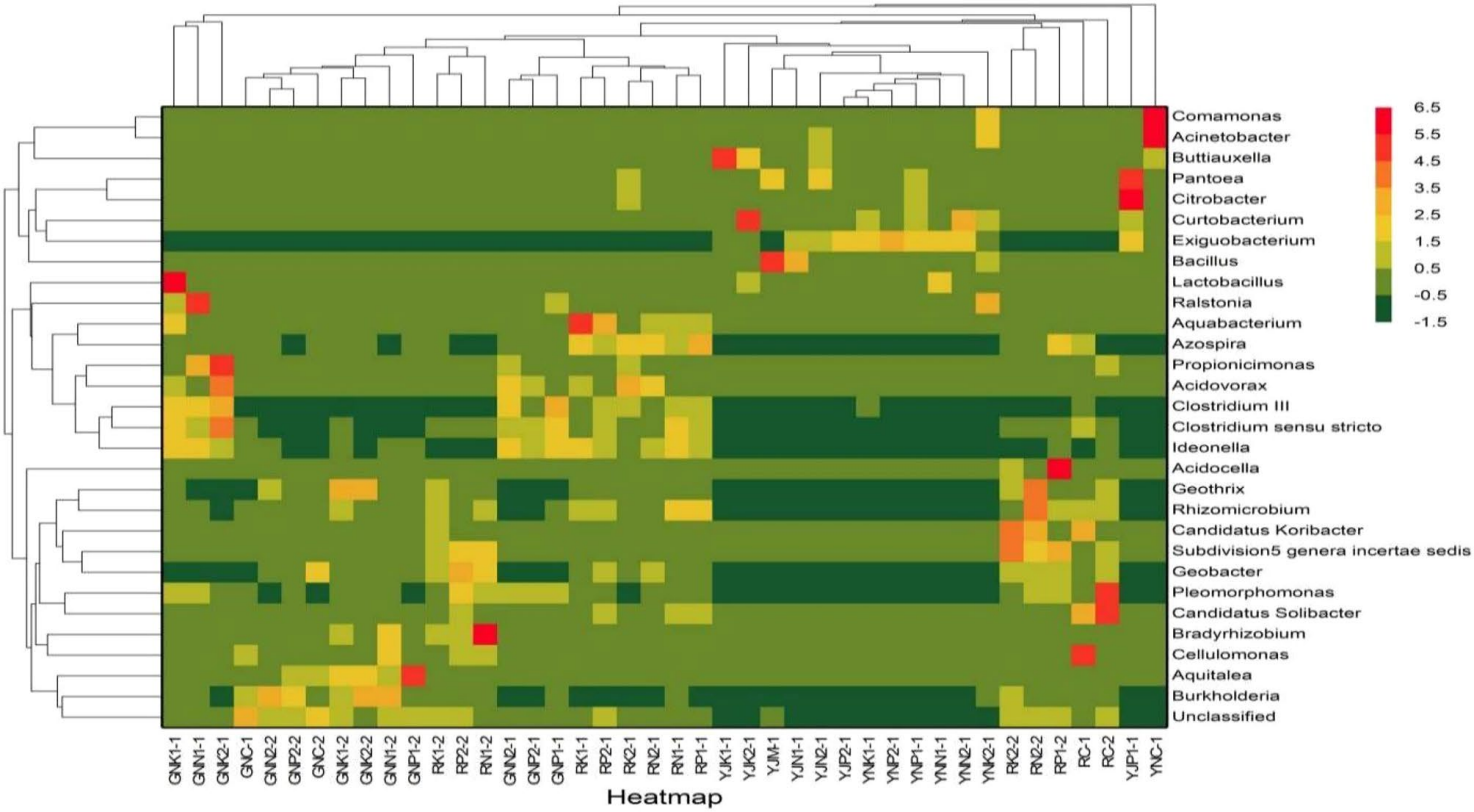
Clustering heat map in Genus level.

Comparing the top 30 bacteria genera in abundance in Fig. 9, it was found that rice straw returning soil affected the community structure of bacteria genera in rice root, rhizosphere, leaf, and phyllosphere. Rice straw returning soil improved the abundance of rice-related bacteria in rice. For example, different rice straw returning amounts and fertilization conditions significantly increased the abundance of *Ideonella*, *Acidovorax*, *Propionicimonas*, *Exiguobacterium, Clostridium III*, and *Bradyrhizobium* in rice roots at the tillering stage, improved the abundance of *Lactobacillus*, *Clostridium *sensu stricto, *Aquabacterium*, *Citrobacter*, *Pantoea* and *Bradyrhizobium* in rice roots at both the tillering and maturity stages, and improved the abundance of *Burkholderia* in rice roots at the maturity stage. Furthermore, rice straw returning increased the abundance of *Ideonella*, *Acidovorax*, *Exiguobacterium*, and *Clostridium III* in rice rhizosphere at the tillering stage, increased the abundance of *Bradyrhizobium*, *Azospira* and *Aquabacterium* in rice rhizosphere at both the tillering and maturity stages, and improved the abundance of Subdivision 5 general *entertae sedis* and *pleomorphonas* in rice rhizosphere at the maturity stage. Rice straw returning soil increased the abundance of *Lactobacillus* in rice leaves at both the tillering and maturity stages, that of *Exiguobacterium Ideonella*, *Acidovorax*, and *Exiguobacterium* in rice leaves at the tillering stage, and that of *Clostridium *sensu stricto, *Aquabacterium*, *Buttiauxella*, and *Curtobacterium* in rice phyllosphere at the tillering stage and maturity stage.

As rice straw returning soil improves the abundance of rice-related bacteria*, Acidovorax, Clostridium *sensu stricto*, Propionicimonas,* and *Clostridium III* in GNK2-1, *Burkholderia* in GNK2-2, *Lactobacillus* in GNK1-1, *Ralstonia* in GNN1-1, a*quitalea* in GNP1-2, A*zospira* in RP1-1, *Geobacter* in RP2-2, a*quabacterium* in RK1-1, subdivision5 genera *incertae sedi*s and *Candidatus koribacter* in RK2-2, *Exiguobacterium* in YNP2-1, *Citrobacter* in YJP1-1, *Buttiauxella* in YJK1-1, *Pantoea* in YJP1-1, *Curtobacterium* in YJK2-1, *Rhizomicrobeum a*nd *Geothrix* in RN2-2, *Bradyrhizobium* in RN1-2, *Pleomorphonas* in RC-2, and *Acidocella* in RP1-2 were significantly higher than that in other samples.

Rice straw returning soil also significantly reduced the abundance of certain related bacteria in rice. For example, it reduced the abundance of *Bacillus* in the phyllosphere of rice at the tillering stage and in the rhizosphere of rice at the maturity stage, resulting in the highest abundance of *Bacillus* in YJC-1 without adding rice straw; the abundance of *Cellulomonas* in rice root and rhizosphere at the tillering stage was greatly reduced, resulting in the highest abundance of *Cellulomonas* in RC-1 sample without adding rice straw; The abundance of *Propionicimonas* in the rhizosphere of rice at maturity stage, *Burkholderia* in the root of rice at the tillering stage, and *Buttiauxella* in the leaf of rice at the tillering stage were also reduced. Furthermore, the abundance of *Acinetobacter* in the leaves of rice reduced; thus its abundance was highest in YNC-1 sample without adding rice straw. Similarly, the abundance of *Comamonas* in the leaves of rice at the tillering stage was greatly reduced; thus its abundance was highest in YNC-1 sample without adding rice straw. Moreover, the abundance of *Candidatus solibacter* and *Geobacter* in the rhizosphere of rice decreased at the maturity stage with the addition of rice straw.

**Figure 10:**
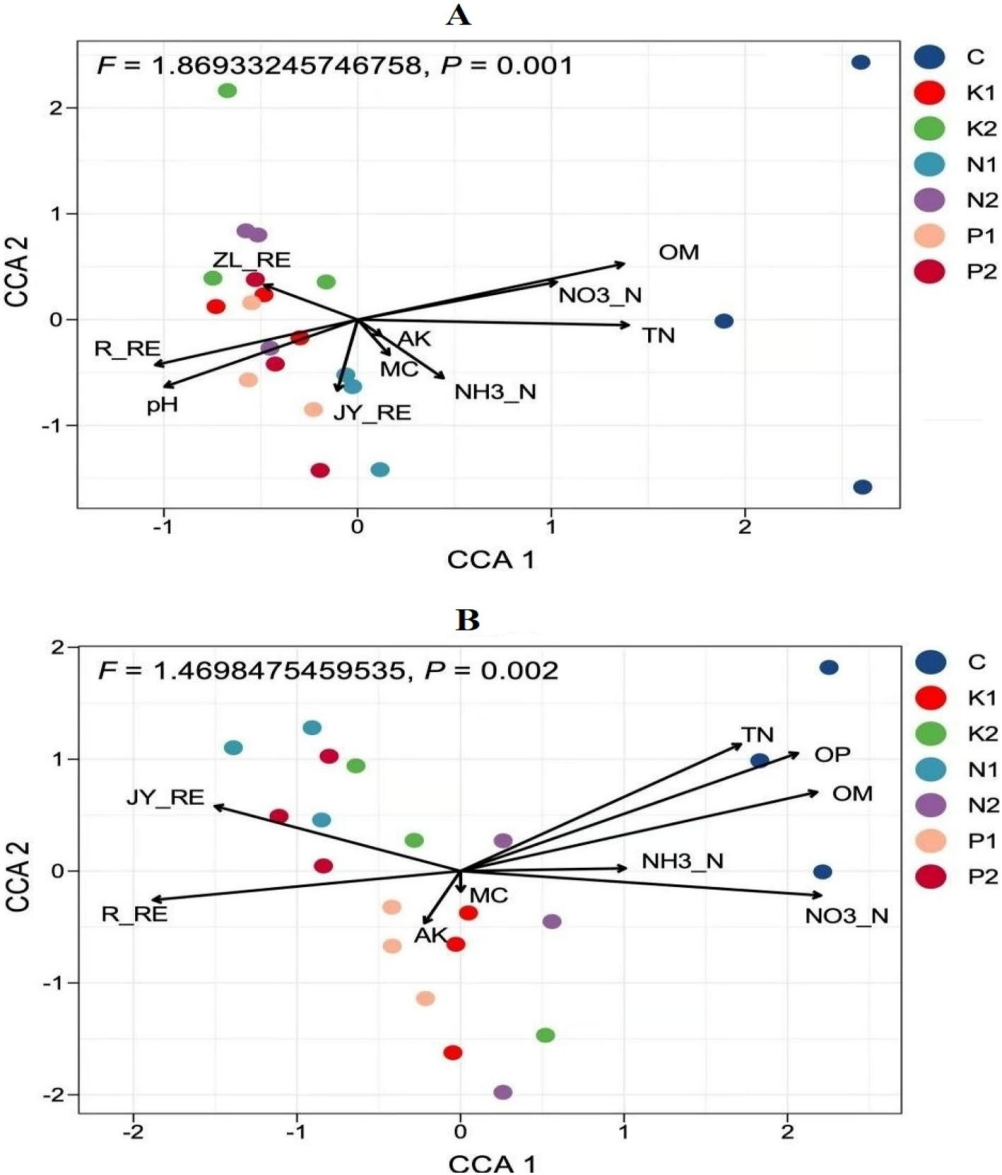
Canonical correspondence analysis (CCA) and CCA based bacterial community variation zoning analysis (VPA) of rice bacterial community. (Figure (**A**) shows the relationship between rice bacterial community and the environmental factors at tillering stage, Figure (**B**) shows the relationship between rice bacterial community and environmental factors at maturity stage; R-RE represents the content of REEs in rice roots, JY-RE represents the content of REEs in rice shoots, ZL-RE represents the content of REEs in rice grains, and MC is the soil moisture content, (**C**) in the figure represent the sample under conditions of no rice straw, that is, the control; N1 and N2 represent represents 1% and 2% rice straw combined with NPK fertilizer, P1 and P2 represent 1% and 2% rice straw combined with NP fertilizer, K1 and K2 represent 1% and 2% rice straw combined with NK fertilizer).

It can be seen from Fig. 10A that environmental factors, including available P, organic matter, total nitrogen, nitrate nitrogen, REE content of the roots, available K, and soil moisture, are important factors affecting microbial communities. The abundance of bacteria in the roots, rhizosphere, leaves, and phyllosphere of rice was positively correlated with available P, organic matter, total nitrogen, and nitrate nitrogen, and was negatively correlated with the REE contents in rice shoots and roots without adding rice straw. The abundance of bacteria in the roots, rhizosphere, leaves, and phyllosphere of rice grown in soil with 1% or 2% rice straw combined with NK fertilizer, and 2% rice straw combined with NPK fertilizer was positively correlated with the content of REEs in shoots and roots, and negatively correlated with available P, organic matter, total nitrogen, and nitrate nitrogen. It can be seen from Fig. 10B that pH, REE content of the roots, organic matter, total nitrogen, nitrate nitrogen, REE content in shoots, soil moisture content, and REE content of the grains are the main environmental factors affecting microbial communities in the roots and rhizosphere of rice. The abundance of bacteria in roots, rhizosphere, leaves, and the phyllosphere of rice grown in soil with 2% rice straw combined with NP fertilizer was positively correlated with the pH and the REE content of roots and grain, and negatively correlated with the organic matter, total nitrogen, and nitrate nitrogen; The microbial community structure in roots and rhizosphere of rice grown in soil without rice straw was positively correlated with the organic matter, total nitrogen, and nitrate nitrogen, and negatively correlated with the pH and REE content of root and grain. The microbial community structure in the root and rhizosphere of rice grown in soil with 1% rice straw combined with NPK fertilizer was positively correlated with the REE content of shoots and the moisture content of the soil, and negatively correlated with available K; it was negatively correlated with the REE content of rice shoots and moisture content of the soil, and positively correlated with available K.

## Discussion

This study shows the different ways of rice straw returning to improve the soil pH, which is not only consistent with the research results of Jin et al. on returning 2.5% rice straw and 1.0% rice straw ash to soil in rare earth mining areas in Longnan and Xinfeng counties of Jiangxi Province, China^2,3^, but also consistent with the results of many other studies on returning rice straw returning soil at home and abroad. Cong et al. reported that rice straw returning accelerated decarboxylation and increased soil pH^38^; Wang et al. found that rice straw returning could effectively improve exchangeable K^+^, Ca^2+^, and Mg^2+^ of soil, which were positively correlated with the soil pH, while exchangeable H^+^ and Al^3+^ of soil were negatively correlated with the soil pH, and their content decreased with the addition of rice straw^39^. Table 1 shows that rice straw returning improves exchangeability of soil K^+^. Therefore, in this study, except for individual cases, irrespective of the stage of tillering or maturity or fertilization method, the pH of 2% rice straw returning soil was greater than that of 1% rice straw returning soil. Because the level of K in rice straw is significantly higher than that of N and P, and the release rate of K from rice straw in the first year is significantly higher than that of P and N, the level of inorganic phosphorus in rice straw returning soil accelerates the depletion and reduces the accumulation of phosphorus. Microorganisms need to consume more nitrogen resources to participate in the degradation of rice straw. Crops compete with microorganisms for nitrogen, resulting in a loss of nitrogen^28,40,41^. Therefore, rice straw returning soil under different conditions in this study led to a significant decrease in soil N and P and an increase in soil K.

In conclusion, rice straw returning soil affects the physical and chemical properties of soil. This effect is related to the amount of straw returned to the field and the method of fertilization 40. Compared with 1% rice straw, the effect of 2% rice straw returning was significant, which reduced the dry weight of rice root, shoot, and grain significantly. The dry weight of rice grain without K fertilizer was the most significant, the dry weight of shoot without P fertilizer was the most significant, and the dry weight of rice root, shoot, and grain with 1% rice straw combined with NPK fertilizer was not significantly reduced. First, the rice straw is returned to the paddy field and soaked in water. Under the action of high temperature, it quickly ferments and expands, which increases the soil gap, hinders the contact between rice roots and soil, and restricts rice rooting and root growth. Second, when rice straw returns soil, microorganisms compete with crops for N, resulting in the loss of N. Third, rice straw returning affects the microbial community and function of rice, resulting in the reduction of rice yield. Rice straw returning soil not only affects the weight of rice roots, shoots, and grains, but also affects the content of REEs. Irrespective of the amount of rice straw returning, the content of REEs in all parts of rice increased, and the effect of the addition of 2% rice straw was more significant. Jin et al*.* showed that rice straw returning reduced the dry weight of rice and increased the content of REEs^42^. Other studies have shown that after adding rice straw to soil, the activity of soil heavy metals increased due to the release of soluble organic matter in a short time, which promoted their migration and accumulation in rice^43–45^. The tillering stage promoted the migration of solid matter from root to shoot, which may be the reason why the content of REEs in the shoot of rice grown in soil with rice straw was significantly higher than that of the control. At the maturity stage, it promoted the migration of heavy metals from shoots to grains, which may be the reason why the content of REEs in grains of rice in rice straw returning soil was significantly higher than that of the control.

Rice straw returning leads to changes in soil pH, C, N, P, and K contents and the bio-availability of soil REEs, and affects the changes in the bacterial community structure and function in rice root, rhizosphere, leaf, and phyllosphere^27^. After rice straw was added to soil under different conditions, the abundance, diversity, and evenness of almost all samples increased significantly, except for the Chao1 index in rice roots that decreased at maturity. Rice straw returning to soil provides more organic carbon, which provides nutrients for bacterial growth, is conducive to their reproduction and growth, and enhances the diversity of the bacteria community in paddy soil^11^. New species may be introduced during rice straw returning, and rice straw returning improves the bioavailability of REEs. These factors may be the reason for the improvement of the richness and diversity of the bacteria community. In this study, rice straw returning soil led to the reduction of soil total nitrogen and ammonium nitrogen. Nitrogen content is an important factor affecting the change in Proteobacteria abundance^5^; therefore, the abundance of Proteobacteria in the roots, rhizosphere, leaves, and phyllosphere of rice at the tillering stage and in the roots and rhizosphere of rice at the maturity stage changed significantly after rice straw returning soil. The content of REEs in soil and plants is the main factor affecting Firmicutes^2,3^. In this study, the addition of rice straw led to an increase in the REE content in rice roots, shoots, and grains, resulting in significant changes in the abundance of Firmicutes in rice roots, rhizosphere, leaves, and phyllosphere at the tillering stage and in the roots and rhizosphere of rice at maturity. Particularly, in rice roots, leaves, and phyllosphere at tillering stage, the abundance of this phylum in the phyllosphere of rice was significantly higher than that in the control. Changes in pH lead to significant changes in the abundance of Acidobacteria in soil^46,47^. After returning rice straw to soil, the soil pH increased, resulting in a significant increase in the abundance of Acidobacteria in the rice rhizosphere at the tillering stage. Studies have shown that rice straw returning to the field will reduce the accumulation of soil organic matter, which can lead to a decrease in Actinomycetes abundance; this may be the reason for the significant decrease in the functional Actinomycetes abundance in rice root at the tillering and maturity stages in this study, which is consistent with many previous studies^2,3,48^. Changes in the type, quality, and quantity of organic matter led to changes in the abundance of Spirochaetes, Bacteroides, Ignavibacteria, Verrucomicrobia, and Chloroflex.

The change of environment leads to the change of bacteria in the host root, rhizosphere, leaf, and phyllosphere, and the change in the bacteria will affect the host. Endophytic bacteria have great influence on rice growth, and their effects on host plants can be divided into beneficial, neutral, and harmful^49^. Beneficial bacteria have the functions of nitrogen fixation, growth promotion, disease resistance, and stress resistance^50,51^. In this study, the dry weight of rice (control) without adding rice straw in the artificial soil was the largest and the content of REEs was the lowest. The abundance of *Bacillus* in the rice phyllosphere without adding rice straw was dozens of times higher than that in rice grown in soil with rice straw. The *Acinetobacter* and *Comamonas* in the leaves and the *Candida solibacter* and *pleomorphonas* in the rhizosphere were significantly higher than those in the rice grown in soil with added rice straw. *Bacillus* is a nitrogen-fixing microorganism, which plays an important role in the regulation of the nitrogen of rice. It can convert gaseous N_2_ into NH^4+^, reduce the leaching loss of nitrate nitrogen, and maintain the nitrogen balance of the ecosystem. This may be an important reason why the soil ammonia nitrogen of sample without adding rice straw was significantly higher than that of other samples. *Bacillus* can inhibit harmful bacteria, pathogens, and other harmful microorganisms. For example, *Bacillus amyloliquefaciens* RWL^−1^ can appropriately increase the production of Gibberellin and salicylic acid in rice and inhibit the production of jasmonic acid and abscisic acid to promote the growth of rice^52,53^. *Acinetobacter* has a strong denitrification ability, which can improve the resistance of plants to heavy metal copper, the ability to remove heavy metal nickel ions, and separate REEs. *Comamonas* also has good degradation ability for a variety of environmental pollutants^54–56^.

At the tillering stage, the abundance of *Exiguobacterium* in the artificial soil with added rice straw was hundreds of times higher than that in the control, and the level in the leaf was dozens of times higher than that in the control. Some studies have shown that the bacterium has a broad-spectrum antibacterial effect on gram-positive and gram-negative foodborne pathogenic bacteria, and also has antagonistic effect on a variety of plant pathogenic bacteria, and shows the ability of nitrogen fixation, phosphate solubilization, and iron carrier generation^57,58^. It may be because the large amount of this bacterium promotes the growth of rice with added rice straw in the later stage, and shortened the gap between the samples and the control. Furthermore, different ways of straw returning to the field had different effects on the bacteria in the root, rhizosphere, leaf, and phyllosphere of rice. When 1% rice straw combined with NPK fertilizer was applied to the field, the abundance of *Exiguobacterium* in the leaves and phyllosphere of rice greatly improved at the tillering stage, the abundance of *Ralstonia* in roots was significantly improved, the abundance of *Bacillus* in rice phyllosphere was second only to the control, significantly higher than those of other treatments, and the *Bradyrhizobium* in rhizosphere was significantly improved at maturity stage. *Exiguobacterium*, *Bacillus,* and *Bradyrhizobium* have the abilities of nitrogen fixation, growth promotion, and stress resistance^53^, which may be an important reason for the high grain dry weight of 1% rice straw combined with NPK. *Ralstonia* is a plant pathogen^59^, which affects the growth of rice, thus accounting for the low dry weight of rice roots and shoots. When 2% rice straw combined with NPK fertilizer was applied to the soil, the abundance of *Exiguobacterium* in leaves and phyllosphere of rice at the tillering stage greatly improved, that of *Ideonella* in rice roots at tillering stage improved, and that of *Rhizomicrobium* and *Geothrix* in rice roots at maturity stage were greatly improved. *Geothrix* promotes the rapid degradation of organic matter, and together with *Rhizomicrobium*, can promote the growth of crops^60^. With the application of 1% rice straw combined with NPK fertilizer, not only was the abundance of *Exiguobacterium* in the leaves and phyllosphere greatly increased at the tillering stage, but also the abundance of *Azospira, Citrobacter,* and *Pantoea* in rice rhizosphere, and the abundance of *Aquitalea* and *Acidocella* in rice roots were significantly increased at the maturity stage. *Azospira* and *Acidocella* in rhizosphere and *Aquitalea* in the root have nitrogen fixation and growth-promoting effects^33,61,62^, which may account for the larger dry weight of rice roots in soil with 1% rice straw combined with NP fertilizer. *Citrobacter* in the phyllosphere of rice may not be conducive to crop growth, and *Pantoea* has been reported to cause rice new bacterial leaf blight^60^; therefore, the dry weight of shoots and grains in soil with 1% rice straw combined with NP fertilizer is lower.

When 2% of rice straw and NP were applied to the soil, the abundance of *Exiguobacterium* in the leaves and phyllosphere at the tillering stage was higher (99.5% and 74.6%, respectively) than that of other similar samples. The abundance of *Geobacterium* in the rhizosphere at maturity was significantly increased, which may be an important reason for the large dry weight of roots and shoots of rice. When 1% rice straw and NK were applied to the soil, not only the abundance of *Exiguobacterium* in the leaves and phyllosphere of rice greatly improved at the tillering stage but also that of *Lactobacillus*, *Acidovorax*, *Clostridium *sensu stricto, and *Aquabacterium* in the rhizosphere and *Buttiauxella* in the leaves greatly improved at the tillering stage. *Lactobacillus* is a beneficial bacterium. *Aquabacterium* and *Buttiauxella* can degrade pollutants and significantly improve the biomass and chlorophyll content of aboveground part and root of Cd-treated plants, which may be the reason for the high dry weight of the roots, shoots, and grains^63^. *Acidovorax* easily causes bacterial diseases. *Clostridium *sensu stricto can produce exotoxins in the environment and affect crop growth^15,64^. When 2% of rice straw was applied to the field with NK, not only was the abundance of *Exiguobacterium* in the leaves and phyllosphere greatly improved at the tillering stage but also that of *Acidovorax*, *Clostridium *sensu stricto, *Propionicimonas*, *Clostridium III*, *Buttiauxella,* and *Curtobacterium* in the leaves and *Burkholderia* in root and rhizosphere of rice at the maturity stage were greatly improved. *Acidovorax*, *Clostridium *sensu stricto*,* and *Curtobacterium* are pathogenic bacteria, they are harmful to plants, which may be the reason why the dry weight of roots, shoots of the rice containing these pathogenic bacteria is lighter than that of other samples. *Buttiauxella* and *Burkholderia* can degrade pollutants and promote crop growth, which may be one of the reason why the dry weight of grains of the rice containing these bacteria is more heavy than that of other samples.

## Conclusion

1. Rice straw returning affects soil pH, N, P, K, organic matter, rice biomass, and REE content. Different rice straw returning amounts and fertilization methods have different effects. Rice straw returning under different conditions improves the soil pH, and the effect of 2% rice straw returning is greater than that of 1% rice straw returning; Rice straw returning soil reduced the accumulation of N, P, and organic matter in the soil and increased the content of soil K, but different ways of rice straw returning had different effects. Furthermore, rice straw returning reduced the dry weight of rice grain, and the reduction with 2% rice straw addition > that with 1% addition, and the reduction with NP combined application > that with NK combined application > that with NPK combined application. Rice straw returning increased the content of REEs in rice, and the increase in the rare earth content by returning 2% rice straw to the soil was greater than that by returning 1% rice straw to soil.
2. Rice straw returning affects the community structure and function of bacteria in rice root, rhizosphere, leaf, and phyllosphere. After rice straw was added to the soil in different ways, except for the decrease in the Chao1 index in rice roots at maturity, the abundance, diversity, and evenness of almost all other samples increased significantly. Rice straw returning reduced the abundance of *Bacillus* in the rhizosphere in rice. The abundance of *Exiguobacterium* of rhizosphere bacterium in rice with the addition of rice straw was hundreds of times higher than that of the control, and the level in the leaf was dozens of times higher than that of the control. Rice straw (2%) returning increased the abundance of harmful bacteria and pathogenic bacteria such as *Acidovorax*, *Clostridium *sensu stricto, *Citrobacter*, and *Curtobacterium.* In contrast, 1% rice straw returning promoted the abundance of nitrogen fixing bacteria, growth promoting, and stress resistant bacteria such as *Lactobacillus*, *Azospira*, *Acinetobacter*, *Bradyrhizobium,* and *Acidocella;* dry weight of rice grains without adding rice straw > weight of rice grains with the addition of 1% rice straw > weight of rice grins with the addition of 2% rice straw.
3. Effective P, organic matter, total nitrogen, nitrate nitrogen, REE content in roots, available K, soil moisture, and other environmental factors are important factors affecting the bacteria community structure in the rice roots, rhizosphere, leaf, and phyllosphere at the tillering stage. The pH, REE content in roots, organic matter, total nitrogen, nitrate nitrogen, REE content in shoots, soil moisture content, and the content of REEs in the grain at maturity are the main environmental factors affecting the community structure of bacteria in rice root and rhizosphere.

## Data Availability

The sequence obtained from the sequencing of the research are available on National Center for Biotechnology Information (NCBI) Sequence Read Archive (SRA) under Project Number: PRJNA874862.
